# Brain basis of communicative actions in language

**DOI:** 10.1016/j.neuroimage.2015.10.055

**Published:** 2016-01-15

**Authors:** Natalia Egorova, Yury Shtyrov, Friedemann Pulvermüller

**Affiliations:** a Medical Research Council, Cognition and Brain Sciences Unit, Cambridge CB2 7EF, UK; b Department of Psychiatric Neuroimaging, Massachusetts General Hospital / Harvard Medical School, 02129, Charlestown, MA, USA; c Centre of Functionally Integrative Neuroscience (CFIN), Aarhus University, Denmark; d Centre for Cognition and Decision Making, Higher School of Economics, Moscow, 109316, Russia; e Brain Language Laboratory, Freie Universität Berlin, 14195 Berlin, Germany; f Berlin School of Mind and Brain, Humboldt-Universität zu Berlin, 10099 Berlin, Germany

**Keywords:** Communicative action, Mirror neuron system, Pragmatics, Social interaction, Theory of mind

## Abstract

Although language is a key tool for communication in social interaction, most studies in the neuroscience of language have focused on language structures such as words and sentences. Here, the neural correlates of speech acts, that is, the actions performed by using language, were investigated with functional magnetic resonance imaging (fMRI). Participants were shown videos, in which the same critical utterances were used in different communicative contexts, to Name objects, or to Request them from communication partners. Understanding of critical utterances as Requests was accompanied by activation in bilateral premotor, left inferior frontal and temporo-parietal cortical areas known to support action-related and social interactive knowledge. Naming, however, activated the left angular gyrus implicated in linking information about word forms and related reference objects mentioned in critical utterances. These findings show that understanding of utterances as different communicative actions is reflected in distinct brain activation patterns, and thus suggest different neural substrates for different speech act types.

## Highlights

• Neural correlates of understanding of Naming and Requesting were studied with fMRI.
• Naming activated the left angular gyrus relevant for referential semantic processing.
• Requests activated the inferior frontal, premotor and temporo-parietal cortices for action sequence prediction.
• Speech act understanding relies on processing of intentions and predicting action sequence structure.

## Introduction

The primary function of human language is to allow efficient communication in social interaction. Yet, the neurobiological mechanisms of this unique communication ability are still poorly understood. Most previous neuroimaging studies focused on structural aspects of language, including the brain basis of word and sentence processing. However, in different communicative contexts, the same utterances are used as tools for different communicative actions, that is, as ‘speech acts’ with different functions, and are likewise understood in such context-dependent manner (Austin, 1962, Searle, 1979, Wittgenstein, 1953). For example, the sentence “My car is here!” can be used to direct the attention of the communication partner to a specific object, to inform the partner about the location of the car, to offer a lift, or to express the speaker's relief that it has not been stolen. It is the situation, context and the social interactive knowledge, that is, *pragmatic* information, that defines the communicative function of the utterance. This study seeks to determine brain activation patterns indexing the comprehension of specific communicative functions by scrutinising the most common speech acts of Naming and Requesting, performed by uttering identical utterances.

A speech act can be defined through a range of features, including a) the linguistic utterance used to perform it, b) the physical setting during the communicative event, c) the action sequences in which the speech act is embedded (i.e., actions preceding and following the speech act), and d) the intentions and assumptions of communicating partners (Asher and Vieu, 2005, Austin, 1962, Bateman and Rondhuis, 1997, Fritz and Hundsnurscher, 1994, Fritz, 2013, Searle, 1969, Van Dijk, 1977). Crucially, with every speech act, a set of likely subsequent actions, or response moves, can be predicted (Fritz, 2013, Pickering and Clark, 2014), so a speech act can be seen as a specific set of action predictions.

To illustrate the relationship between speech acts, i.e., their linguistic and situational components, intentions and assumptions, and predictive sequence structures, consider a situation, in which 2 people (called here a Speaker and a Partner) sit at a table with several objects in front of them: a glass of water, a carton of juice and an apple (the physical setting for the communicative situation). If the Speaker utters the single word utterance “water” while pointing at a glass of water on the table, there are several ways in which this can be understood by the Partner. For example, in a communicative situation when the Partner had initially inquired “What are these called?”, the Speaker by uttering “water” will perform the speech act of Naming. As both the Speaker and the Partner know the language, this interaction can be seen as similar to, for example, a question in an exam and an answer to it. In contrast, if the Partner first opens the sequence by saying “What can I get you?”, then the same word “water” will be understood as a Request. This simple sequence is now used in a way similar to ordering a drink in a bar. In the two hypothetical cases above, the critical linguistic utterance (“water”) and the physical setting (the table, the glass and other objects, the two communication partners opposite each other) are identical. At the same time, the communicative function and the features of context critical for defining it, especially the assumptions and intentions of both communicating partners, as well as the expected action sequences, noticeably differ. For the utterance to count as an instance of Naming, the Speaker needs to assume that the used utterance is appropriate to refer to the object in question, and that he/she pronounces it properly. Following the utterance performing the speech act of Naming, the Speaker can, for instance, point at the Named object, the Partner can ascertain the appropriateness of the utterance by repeating it, correcting it, or asking the Speaker to repeat it. What appears of special relevance to Naming is the semantic referential link between the specific object and the word denoting it. In contrast, Requests involve additional *action knowledge* (e.g., that an object needs to be manipulated) and social or *theory of mind knowledge* (e.g., recognising the Speaker's desire to obtain the object).

Whilst the investigation of brain mechanisms of social-communicative action understanding is a relatively new field of study, sometimes called neuropragmatics, some theoretical and experimental work has already been done (Bara et al., 1997, Hirst et al., 1984, Holtgraves, 2012, Stemmer et al., 1994). Neuropsychological research on brain lesions eliciting deficits in processing pragmatic information provided evidence that cortical areas in both cerebral hemispheres can be crucial for understanding even the basic speech acts, such as assertions, commands, and Requests (Soroker et al., 2005). Furthermore, some recent studies in healthy volunteers used neuroimaging methods to investigate the brain basis of the so-called indirect speech acts, for example when someone Requests that the window be opened by saying “It is hot in here” rather than directly saying “Open the window” (Van Ackeren et al., 2012); see also Basnáková et al. (2014). Other recent work has focused on the neurophysiological basis of turn taking (Bögels et al., 2015) and the partner knowledge in language processing (Rueschemeyer et al., 2014). In the present study, we addressed the previously under-studied question of whether comprehension of basic social communicative actions such as Naming an object or Requesting it, placed in otherwise identical settings, would be reflected in different neurometabolic signatures.

The current experiment manipulated speech act types whilst keeping constant a range of relevant features of the stimulus materials and communicative context. Not only were the same utterances used to perform different speech acts, we also meticulously matched all visual, acoustic and linguistic features of the stimulation, assuring, for example, that the same physical setting was established with the same two communicating partners and the same sets of objects being present in different communicative contexts. Furthermore, the sequences and linguistic contexts of each speech act type, Request and Naming, were matched.

## Experimental predictions

The summarised linguistic and neurobiological considerations offer specific hypotheses about the brain loci for speech act processing. As Naming puts an emphasis on the link between a name and its semantically related reference object, especially strong activation is predicted in this condition in areas relevant for linking linguistic and visual object representations — in the middle temporal cortex (Damasio et al., 1996, Pulvermüller and Fadiga, 2010) and the left angular gyrus (Binder et al., 2009, Geschwind, 1970). Stronger activation for Requests compared with Naming actions can be hypothesised in the areas subserving action and social interaction knowledge. The areas linked to action performance, perception and prediction are in the fronto-central sensorimotor cortex (Pulvermüller and Fadiga, 2010, Pulvermüller et al., 2014) and temporo-parietal cortex (Fogassi et al., 2005, Noordzij et al., 2010, Saxe, 2009) and include the human homolog of the mirror neuron system found in macaques and distributed across premotor, inferior frontal and anterior inferior parietal cortex (Rizzolatti and Fabbri-Destro, 2008, Rizzolatti and Sinigaglia, 2010). Cognitive processing of others' assumptions and intentions may engage what is commonly labelled the ‘theory of mind’, or ToM, system, which includes the medial prefrontal cortex, anterior cingulate and the temporo-parietal junction (Canessa et al., 2012, Frith, 2007, Spunt et al., 2011, Van Overwalle and Baetens, 2009).

Previous studies of speech act processing showed a difference between speech act types in neurophysiological activation patterns revealed by EEG and MEG (Egorova et al., 2014, Egorova et al., 2013), but, due to the known spatial imprecision and uncertainty of these methods (Hamalainen et al., 1993), it is necessary to employ more precise localisation tools to track focal activation changes reflecting communicative action and interaction processing. Therefore, the current study focused on neurometabolic patterns revealed by fMRI to determine the cortical foci indicative of speech act comprehension in the referential semantic network, the action-semantic/mirror neuron network, and/or the ToM network.

## Materials and methods

### Participants

Twenty healthy native English volunteers took part in the study. The data from 2 participants were discarded due to excessive movement artefacts. The data from the remaining 18 participants (10 female) with mean IQ score M = 36.6 (range 28–44) measured by the Cattell Culture Fair Test, Scale 2 Form A [Institute for Personality and Ability Testing, 1973 (Cattell, 1971)] , mean age 27 years (range 18–41) were analysed. All the participants were right-handed as assessed with the Edinburgh Handedness Inventory (Oldfield, 1971), with mean laterality coefficient of 90.2 (range 60–100). The study was approved by the Cambridge Local Research Ethics Committee (Cambridge, UK). The experimental manipulations were explained to the participants and informed consent was obtained prior to the start of the experiment.

### Stimuli

The stimuli consisted of 16 experimental video scenes showing two persons (a “Partner” and a “Speaker”) sitting at a table with 12 objects in front of them. Each scene appeared four times during the experiment, resulting in a total of 64 speech act trial sequences. Each trial sequence (Fig. 2) started with a *context sentence* uttered by the Partner, which set the stage for the subsequent speech act, for which each of the *critical utterances* (names of 5 out of 12 objects on the table) uttered by the Speaker was used. Depending on the context sentence, the critical words were used either to Name or to Request items on the table. Sequences of repeating speech acts in the experiment modelled repetitive use of the same speech act type, as commonly used, for example, when placing orders in a restaurant. Each word appeared in both Naming and Requesting conditions. Following the critical utterances, 5 nonverbal actions corresponding to the speech act type of the trial sequence ensued, pointing at the mentioned objects in the Naming condition, and handing the objects over in the Requesting condition. Context sentences and subsequent actions were added to embed the speech acts in a natural context and to make sure, at the same time, that Naming and Request speech act trials were completed in a similar fashion. (Note that Naming without pointing and Requesting without subsequent expected action may be evaluated as unnatural; furthermore, the isolated presentation of utterances outside action contexts may even undermine the status of these utterances as speech acts of a specific type and their communicative relevance.) In addition to the critical trials with their Context–Utterance–Action sequences (32 for each speech act type), we added several control trials (16 per speech act type) in which Context sentences were followed by silent still Face stimuli (5 face pictures appeared instead of words). This type of trial made it impossible to predict upcoming speech acts with certainty, as the context sentences did not always conclude with a Naming or Requesting action. Six individuals (all native British English speakers) were used as actors in recording the videos. Two of them (one female) acted as Partners, and four (two female) were Speakers. Their positions in relation to each other (left–right) were fully counterbalanced.

Six sentences matched on the number of words and complexity and representing different syntactic types (interrogative, imperative) were used to provide the context for the speech acts. Three introduced the context for the speech act of Naming (e.g., “What are these called?”) and three for the speech act of Request (e.g., “What can I get you?”). These sentences were pseudo-randomly used in all trial sequences.

160 monosyllabic nouns from various semantic categories – referring to food items, tools, animals, clothes and other everyday objects – were used as stimuli. Their psycholinguistic features (Table 1) were obtained from the CELEX database (Baayen et al., 1993); furthermore, their semantic properties were rated (7-point Likert scale) by a separate group of 10 native speakers of English.

Finally, all trials were evaluated by 5 native English speakers of similar age and educational background to the ones who took part in the experiment. Each subject watched a set of 20 videos (10 of each type) and described “what they saw and heard and what they understood the Speaker and Partner did” in given trials. There was agreement on the judgement of scene content, especially on that the Speaker either Named (labelled, tagged, etc.) or Requested (asked for, solicited, etc.) an object in the respective trials. Thus, despite the usage of the same utterances, observers reliably understood the speech acts differently in the two context types, in accord with the experimental design.

### Presentation procedure

The experiment started with visually presented instructions. The participants were informed that they would see videos of two people interacting, and that one of them would ask the other to name the objects on the table, or to ask for these objects. The different trial sequences appeared in a pseudo-randomised order.

The participants were instructed to carefully watch the scenes showing communication between two people and were told that they would be tested later to check if they paid attention to the content of the videos. Subjects were not told to memorise scenes, utterances, persons or objects. There was no button-press or other motor task during the experiment, as it would elicit motor activation that could contaminate the motor system activation, which is especially important in this experimental context, as language-related motor-system activity is predicted to occur in response to speech acts as such.

After the participants came out of the scanner, they were given a list of 40 words, which contained both words mentioned in the experimental video and previously unencountered foils. The task was to mark the words they remembered as present in the videos, where they had been part of critical utterances. Performance in the behavioural task was assessed by calculating d-prime values for all participants. The d-prime statistic measures the performance in discriminating between targets and non-targets, by taking into account both hit rates and false positive rates and thus controlling for any possible response bias. In this experiment, the d-prime calculation was based on the discrimination between the words that appeared both in the experimental videos and on the list (targets) and the words that did not appear in the experimental videos but were present on the list (non-targets).

### Image acquisition and analysis

The experiment was run at the MRC Cognition and Brain Sciences Unit, Cambridge (MRC-CBSU) using a 3 Tesla Siemens Tim Trio scanner (Siemens Medical Solutions, Erlangen, Germany). MRC-CBSU continuous “quiet” EPI sequence with a substantially reduced acoustic scanner noise (Peelle et al., 2010) was used with TR = 2.656 s, TE = 44 ms, acquiring 32 descending 3 mm thick slices in axial oblique orientation, slice gap of 25%, FOV of 192 mm × 192 mm, flip angle of 83°, and bandwidth of 1220 Hz/Px. The experiment was programmed in E-prime 2.0 (Psychology Software Tools, Pittsburgh, PA) and the scenes were projected onto a screen, visible from the scanner via a mirror, whilst the audio-visually presented context sentences and words were delivered via noise-insulated headphones.

Pre-processing and all analyses were done using the Statistical Parametric Mapping software (Wellcome Department of Cognitive Neurology, London, UK). The pre-processing included slice time correction, reorientation to correct for motion, spatial normalisation to the standard MNI (Montreal Neurological Institute) template, smoothing with an 8 mm full-width at half-maximum Gaussian kernel and high-pass filter.

General linear models were used for the fixed effects analyses on each subject's data. We focused on the sequences of 5 critical utterances appearing after the context sentence which were modelled as one utterance (including 5 utterances each) trial. The blocks were convolved with the canonical hemodynamic response function (HRF). Other types of events (e.g., Context sentences, Action blocks, as well as Face blocks) were also modelled. These other types of events were crucial to fulfil the conditions for a successful speech act (context sentences introduced the situation, control face blocks prevented the context sentences from unambiguously predicting upcoming speech acts; action blocks were necessary to preserve the natural speech act structures).

Group level random effect analysis with the speech act condition as a within subject factor was carried out for the whole brain volume in a factorial design. This analysis was used to determine which brain areas were predominantly engaged in the Naming and Request conditions. The results from the whole-brain analysis were corrected for multiple comparisons using false discovery rate (FDR) correction at p < 0.05 for all brain areas.

Small volume correction (SVC) was used on a set of a priori regions of interest (ROIs), based on the function–anatomy correlations established independently in previous studies (Binder et al., 2009, Van Overwalle and Baetens, 2009). Spheres with an 8 mm radius around the MNI coordinates taken from representative studies and reviews or the centre of ROI mass in the Automated Anatomic Labeling (AAL) atlas (Tzourio-Mazoyer et al., 2002) were used for SVC; the coordinates are provided in Table 4. Specifically, these a priori defined regions included

– the *action-semantic and mirror neuron areas* — left IFG (Caplan, 2006, Fadiga and Craighero, 2006, Pulvermüller and Fadiga, 2010, Rizzolatti and Craighero, 2004), bilateral PMC (Hauk et al., 2004, Kiefer and Pulvermuller, 2012, Willems et al., 2010b), left aIPS (Fogassi et al., 2005, Hamilton and Grafton, 2006, Ramsey et al., 2012), right pSTS (Materna et al., 2008, Noordzij et al., 2010, Proverbio et al., 2011, Redcay et al., 2012) as a multimodal integration area especially relevant for speech (Calvert, 2001, Hein and Knight, 2008, Szycik et al., 2009);
– the *theory of mind regions* — bilateral TPJ (Saxe, 2009, Scholz et al., 2009), medial PFC (Canessa et al., 2012, Frith, 2007, Spunt et al., 2011, Willems et al., 2010a), as well as bilateral anterior cingulate (Frith, 2007, Gallagher and Frith, 2003) for comprehension of Requests;
– and *referential-semantic brain region*, the left angular gyrus (Binder et al., 2009, Seghier et al., 2010) for understanding of the speech acts of Naming.

A similar set of brain areas (bearing in mind the lower resolution of the method) has been previously identified in our MEG study of Naming and Requesting with visually presented words (Egorova et al., 2014). All SVCs were considered significant at p < 0.05_FWE-SVC_. We also attempted at extracting a set of ROIs traditionally linked to semantic processing. These regions, in the inferior and middle temporal cortex produced very little activation in the present study and no significant differences between conditions. We therefore omit them here.

In addition to the SVC analysis of activity in a priori defined ROIs (8 mm radius each), we extracted average BOLD signals using the MarsBar utility (Brett et al., 2002) to compare average activation of the action and theory of mind areas between speech act conditions. To this end, a repeated measures ANOVA with factors System (Action-semantic vs. Theory of Mind) and Speech act (Naming vs. Request) was performed, followed by additional ANOVAs performed for each system separately, using the factors ROI (5 levels) and Speech act (Naming vs. Request) within each of the systems. Huynh–Feldt correction was applied where appropriate.

In addition, we investigated the brain areas showing significantly stronger haemodynamic responses in the speech act conditions, where spoken words were presented together with faces, contrasted against the Face blocks, where faces were shown without linguistic stimuli (contrast “Words > Faces”), using whole-brain analysis (p < 0.05 FDR).

## Results

### Behavioural results

D-prime values were calculated as a measure of participants' performance. For all subjects, d-prime values were high (mean 2.6, range 1.06–3.58), indicating good stimulus item recognition as well as compliance with the task and attention to the experimental scenes.

### Imaging results

Comparison of all speech act conditions together against control face trials (face without speech act) yielded activation in the bilateral superior temporal cortex, including the auditory cortex, consistent with speech stimulation. In addition, middle and inferior temporal activation was present, likely due to object-related referential expressions used in all speech acts. Hippocampal activity was also observed, consistent with the memory load imposed by the experimental context. The right angular gyrus/right TPJ (Carter and Huettel, 2013) was found active, which is consistent with the general involvement of the theory of mind processes in all speech acts under examination (Table 2).

In the critical analyses, which directly compared speech act conditions with each other, the contrast “Request > Naming” showed significant differential activation (p < 0.05, FDR-corrected) in a number of cortical areas in both hemispheres (Table 3), including the middle and superior occipital areas. There was a significant differential activation in the left inferior frontal region, bilateral premotor and right posterior temporal regions, including right pSTS. The activation for this contrast is shown in red in Fig. 3A. On the contrary, the “Naming > Request” contrast did not produce any significant activation at the FDR-corrected significance threshold of p < 0.05.

Small volume corrected ROI analysis performed for the regions selected a priori following the previous literature (see Materials and methods), revealed several significant clusters for the action system ROIs — left IFG, bilateral PMC, left aIPS, right pSTS, but no superthreshold clusters for the ToM ROIs in the “Request > Naming” contrast. The opposite contrast, “Naming > Request” revealed only a non-significant trend for the left AG activation (Table 4).

To test the hypothesis that both the Action and the ToM systems contribute to the processing of speech acts (especially Requests), we performed a repeated measures ANOVA on the averaged activation obtained per condition from the same ten pre-defined ROIs (on the basis of existing research results), five in the action-semantic and five in the ToM system, respectively. The results showed a significant interaction of Speech act type and system [F_(1,17_) = 6.955, p = 0.017]. To further understand this interaction, we performed a repeated measures ANOVA on the ROIs by Speech act type within each system, and found a main effect of Speech act type [F_(1,17)_ = 8.845, p = 0.009] in the Action system ROIs (Request > Naming) but no significant effects or interactions in the ToM system ROIs. Across all action system ROIs, activation was significantly greater for Request than for Naming, Fig. 3C. Pairwise comparisons (Naming vs. Request) for each of the ROIs in the Action system were significant, corrected for multiple comparisons with FDR adjustment, p < 0.047.

## Discussion

**Fig. 1:**
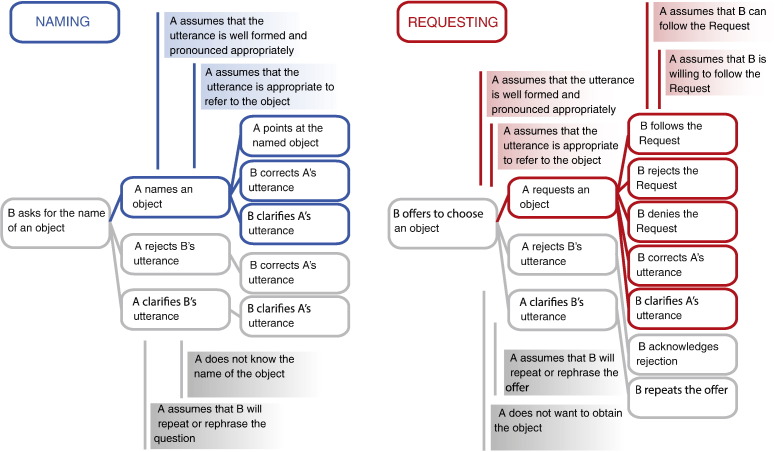
Action sequence schemas of the speech acts of Naming (left) and Requesting (right) show typical actions following these speech acts and the intentions and assumptions (in shaded boxes) associated with them.

**Fig. 2:**
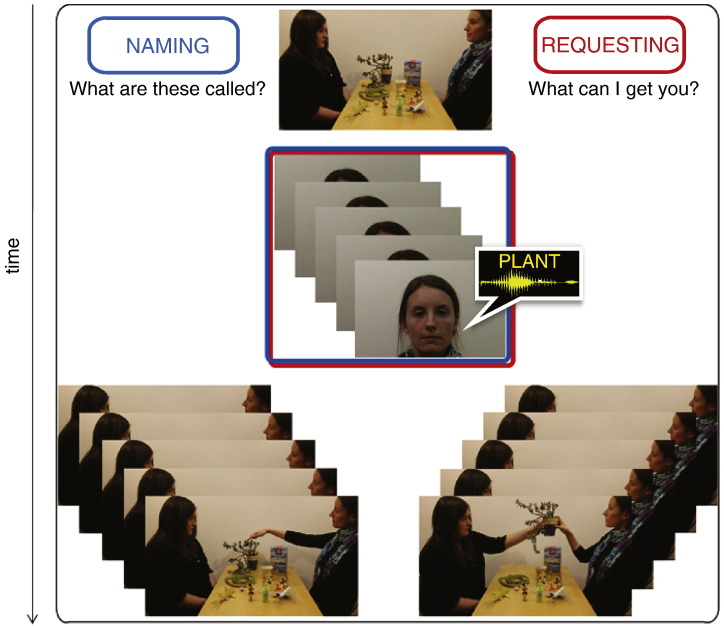
Schematic illustration of the trial sequence in the fMRI experiment. A trial sequence started with a display of objects and communicating actors. A context sentence (e.g., “What are these called?” in the Naming condition, or “What can I get you?” in the Requesting condition) was uttered by the Partner. Following this, a series of 5 scenes was shown, in which the Speaker's face appeared together with the critical spoken utterance which served for Naming vs. Requesting an object (note that the words were identical for both speech acts, see the Materials and methods section). The word scenes were followed by a series of 5 action scenes, involving the objects mentioned in the word utterances (handing over an object in the Requesting condition or pointing at it in the Naming condition). Each context sentence, word, face and action video clips lasted about 2 s.

The comprehension of different speech acts performed with the same words in closely matched interactive settings led to significantly different brain activation patterns. Requesting objects compared to Naming them was characterised by stronger activation, especially in the left inferior frontal (IFG), bilateral premotor cortex (PMC), as well as the left anterior inferior parietal cortex (aIPS), right posterior superior temporal sulcus (pSTS) and adjacent occipital cortex. In turn, Naming tended to more strongly activate the left angular gyrus (AG) in the posterior parietal cortex, compared with Requesting. These differences in cortical activation cannot be attributed to either the critical linguistic stimuli, as these were kept constant across tasks, or to the general stimulus setup (including perception of actions and objects), as that was strictly matched. Therefore, they are most likely related to the specific communication contexts in which the linguistic utterances were used. Understanding a Request implies knowledge about the rich sequence structure of this speech act, that is, about the set of manual or verbal actions that typically follow Requests in real dialogues and therefore can be predicted (Alston, 1964, Pickering and Garrod, 2009, Van Berkum, 2010) from the critical utterances used in the Request context. We hypothesise that the motor system and the left inferior frontal gyrus are the brain regions supporting prediction of a rich set of possible but alternative response actions (see Fig. 1 for illustration) forming the mental basis for Request processing.

### Distinct brain correlates for understanding speech act types

As demonstrated by the results, when subjects observe and understand communicative interactions between the Speaker and the Partner, different brain activation patterns emerge for comprehending the communicative speech acts of Requesting and Naming. Note that this paradigm did not use an active speech production task, where subjects would perform the relevant speech acts themselves, nor were subjects directly addressed in the dialogue sequences or had to respond to the crucial speech acts. Our intention was to minimise the likelihood of any movement artefacts in this experiment whilst focusing on such relevant aspect of communicative competence as the capacity to *understand* communicative interaction between other individuals, as it is required, for example, to watch a TV interview or follow a conversation between two friends. We instructed our subjects to focus their attention on understanding the communication between the Speaker and the Partner; in order to keep them motivated and attentive throughout the experiment, we told them that a test (the nature of which remained unspecified until after fMRI scanning) would be administered after the experiment.

In investigating speech act understanding, we focused on two actions, Naming and Requesting, which are key speech acts in communication theory and have been used to illustrate pragmatic language function (Wittgenstein, 1953, Baker and Hacker, 2009). Naming can be seen as an elementary language function for which the referential relationship between words and objects is manifest. Assumptions about the Speaker and the Partner are involved insofar as the labels used and their object links are considered part of the language knowledge and common ground of both. Correspondingly, Naming actions in real life, for example in the context of questions in a foreign language test, could be followed by corrections (as indicated in Fig. 1). In contrast, the Request actions, compared with Naming in the present experiment, imply a substantially richer communicative context, in which each Request can not only (as any action) be followed by corrections, but is firmly associated with the specific prediction that the partner hands over the Requested object or, alternatively, refuses or rejects the Request (which, in turn, can be done verbally, by gesture or facial expression). This richer set of action predictions, which is characteristic of Requests according to pragmatic theories, was reflected in the activation of the left inferior frontal, left inferior parietal and bilateral premotor cortices. These regions are also part of the cortical system for motor and action processing, where, in the monkey cortex, mirror neurons are frequently found (Rizzolatti and Craighero, 2004, Rizzolatti and Fabbri-Destro, 2008). The stronger activation during Request understanding, compared with that of Naming, may therefore reflect the prediction of actions such as manual handing over the Requested object or explicit verbal or manual rejection of the Request. The localisation of the Request activation in the inferior frontal Broca's region (pars triangularis, BA45) and dorsolateral premotor cortex (hand representation) is consistent with this interpretation and confirms predictions of the action perception model discussed in the Introduction section. The anterior inferior parietal cortex is also part of the action and mirror neuron system, so that its stronger activation during Requests is also in line with the action prediction perspective. A degree of arbitrariness exists for the left temporo-parietal cortex, where part of the angular gyrus, tended to be activated more strongly by Naming. A mirror neuron perspective might have suggested the opposite but the well-known function of the left AG as a hub for semantic processing (Binder et al., 2009, Seghier et al., 2010) might tentatively explain the stronger activation tendency during the speech act, emphasising the referential word–object link. Other areas also known to contribute to referential knowledge, such as anterior temporal cortex, did not show differential activation for the speech acts under investigation. Key sites for ToM processing, such as the right TPJ, were active during processing of both speech act types.

### Requests and the action prediction system

Several studies suggested that, due to mirror mechanisms linking together action and perception circuits, the action system of the human brain is engaged when people observe the actions of others (Decety et al., 1997, Fadiga et al., 1995, Ramsey and Hamilton, 2012, Van Overwalle and Baetens, 2009). The reason for this activation may be an intrinsic link between action and perception mechanisms due to pre-established wiring and associative learning (Pulvermüller and Fadiga, 2010) and the resultant mechanisms for predictive processing of actions based on sensory input alone (Kilner et al., 2007). In this view, the action-semantic and mirror neuron systems may also contribute to the understanding of communicative actions. Consistent with the prediction hypothesis, mirror neurons in the inferior frontal and parietal areas were shown to process information about action goals and intentions motivating motor acts (Fogassi et al., 2005, Ramsey et al., 2012). Therefore, it is possible that the activations observed in the inferior frontal, premotor cortex and anterior inferior parietal cortex contribute to the recognition of communicative intentions and goals and in the prediction of subsequent actions, which is especially important when processing Requests. Note that the link between actions and their goals or intended consequences is part of the predictive sequence structure characterising speech acts (Fig. 1). For example, the goal of obtaining the Requested object is manifest in the expected Partner action of handing over the object (as discussed in the previous section). Therefore, the strong involvement of the action and mirror neuron system in frontal and parietal cortices when understanding Requests, observed in the current experiment, may reflect two inseparable aspects of speech acts: the processing of communicative intentions and goals characterising the speech act and the *predicted sequence structure of typical response actions*. On the other hand, it is also possible that the experimental setting, which encouraged the expectation of a manual action of handing over the target object, led to activation of the mirror neuron system during Requests. However, this interpretation suggests a comparably strong sensorimotor involvement when expecting pointing gestures, which regularly followed the Naming actions in our experimental design, and therefore fails to account for the difference between Naming and Requesting. Therefore, we tend to favour the alternative possibility that the speech act of Requesting, characterised as a socially established goal-directed intentional activity embedded in a sequence of communicative actions and requiring specific shared knowledge between communication partners, activates a specific type of action prediction circuit (Pulvermüller and Fadiga, 2010, Pulvermüller et al., 2014). This speech act circuit appears to be distributed over fronto-parietal areas. At the cognitive level, its activation may imply the computation of an action tree (see Fig. 1) that includes the future actions and outcomes predictably tied to the knowledge about the socially established communicative action^1^.

In addition, increased activation in the bilateral temporo-occipital cortex for Requests compared with Naming was observed, although it had not been explicitly hypothesized previously. We suggest that this stronger activation in areas related to visual information processing may also be best explained in the action prediction context. The richer predictions implicated by Requests, in addition to being manifest in expectations of motor activity, may also lead to expectations of the upcoming visual input. Brain correlates of visual expectations and predictions are known to modulate activity in the occipital and middle temporal cortex (Cléry et al., 2013, Den Ouden et al., 2009). Speech act-related modulation of activity in these areas observed here may therefore provide a candidate explanation in terms of predictive coding (Kilner et al., 2007). An alternative explanation may be offered in terms of memory processes or imagery, which our subjects may have engaged in attempting to memorise features of the presented scenes. However, such general memory or imagery related activation could not easily explain why Requests led to stronger activation than Naming, because memory and imagery should equally be possible with both. In addition, the pattern of brain activation observed in the Request condition appears quite different from typical memory activation patterns, normally spread across the dorsolateral prefrontal cortex and the hippocampal formation (Ishai et al., 2000). These activations were absent in the present contrast; instead, we observed premotor and anterior inferior frontal activation differences, which are more in line with the action prediction hypothesis. Finally, the hippocampus was equally engaged during both types of speech acts, as the results suggest, which further discourages a *differential* memory interpretation. However, memory processes are engaged by all speech acts, as memory is necessary to relate utterances to their preceding context.

Some features of our experimental communicative settings may seem atypical at a first glance, but, in our view, represent one relevant choice amongst a set of equal alternatives, whilst helping to accommodate our experimental questions within confinements of fMRI recording settings. First, following each context sentence, critical speech acts were always presented consecutively in groups of 5 actions of the same type. As mentioned in the Materials and methods section, this mirrors communication typical for, e.g., placing orders in restaurants or shops. Furthermore, because the complex set of actions that may follow specific speech acts in dialogues (see Fig. 1) cannot be captured by a single prediction, our design realised different response options to the context sentence; however, alternative action options were not realised upon Naming and Requests, which were always followed by the respective ‘typical best’ response action (pointing or handing over). This simplification of the action tree should not distract from the fact that, even though alternative response expectations to critical utterances were not encouraged in the present experimental context, speech act understanding can be described in terms of action predictions acquired in the broader context of language learning in social interaction (Alston, 1964, Fritz, 2013, Tomasello, 2010). An MEG experiment similar to the present one realised several different response types to the critical utterances (Egorova et al., 2014) and showed that the stronger activation of the motor system in Request contexts was also present with such richer communicative embedding. Future research may fruitfully focus on the influence of the relationship of general action predictions characterising a socially established communicative action and the particular predictions enforced by specific experimental settings.

A further possible criticism addresses the linguistic structural level. According to some linguistic theories, single word utterances such as the ones used as critical stimuli, are conceptualised as ellipses, i.e., short forms derived from more elaborate structures by omitting some of their syntactic constituents, especially when they are supplied by the context (Matthews, 2007) (for example see (Claus, 2015) for recent experimental evidence on syntactic gapping). In this view, single words would first need to be expanded into full sentences during comprehension, e.g., “water” into whole sentences such as “Please give me water”, or “The object to which I point is water”. A strong ellipse extension perspective could suggest that, what we consider as speech act differences might in fact be attributable to syntactic differences in presumed expanded ‘deep structural’ representations. Assuming that such expanded structures would come with subject, predicate and object, this approach might possibly lead to a straightforward explanation of differential involvement of the inferior frontal cortex. It, however, would not explain differential engagement of the precentral cortex and the angular gyrus, because syntactic processing seems to be bound to different brain structures. Moreover, a range of possible expanded versions are available for each context (for Request: “Give me X”, “Please give me X”, “I would like to ask you to take X and hand it over to me”; likewise, for Naming: “This is X”, “I call this X”, “The name of this object is X and I would suggest we both use this word to speak about it” etc.) and thus the choice of specific ‘deep structural’ representations appears to a degree arbitrary for our present contexts. For these reasons, expanding elliptical structures to full sentences does not seem to provide a convincing explanation for why the observed brain structures become differentially engaged for Requesting and Naming. Furthermore, whilst the fMRI technique does not provide satisfactory temporal resolution, our previous EEG and MEG work suggested very rapid engagement of these systems (within ~ 200 ms), thus providing an argument against any elliptical expansion potentially requiring more processing time. For further arguments against the idea that single word utterances are necessarily elliptical, see (Wittgenstein, 1953 and Baker and Hacker, 2009).

Although neuroscience studies of communicative actions are sparse, some important research has recently been conducted in this domain (see Introduction) and we should therefore relate our present findings to some of this earlier work. A recent study (Van Ackeren et al., 2012) looked at statements, such as “It is hot in here” used either for Informing others of the ambient temperature or as indirect Requests to open the window. The visual context that accompanied the linguistic utterances differed between the Informing and Requesting conditions — e.g., images of a desert landscape or a window, respectively. Stronger brain activation for indirect Requests appeared in the fronto-central action system as well as in the parietal areas previously related to mirror neuron intention understanding (Iacoboni et al., 2005, Rizzolatti and Craighero, 2004). A second set of increased brain responses to indirect Requests appeared in the medial prefrontal cortex and bilateral temporo-parietal junction, regions typically observed in theory of mind (ToM) processing, for example when people think about others' knowledge and intentions (Van Overwalle and Baetens, 2009). However, as this previous study altered communicative function (Request vs. Informing) together with directness (indirect vs. direct), it is difficult to draw conclusions on which of these factors – communicative function, (in)directness, or both – explain the observed differences in brain activation. Similarly, Basnáková et al. (2014) investigated the difference between answers given directly as statements following questions (“How is it … to give a presentation?” followed by “It's hard to give a good presentation”) or indirectly by justifying the implied answer (e.g., “How did you find my presentation?” followed by “It's hard to give a good presentation”). Stronger brain activation to indirect justifications than to direct statements was seen in areas related to ToM (medial prefrontal cortex, right temporo-parietal junction), emotion (anterior cingulate, anterior insula) and action processing (inferior frontal cortex, SMA). In this study, indirectness also came with a change in speech act type (statement vs. justification), so that a degree of openness remains for the interpretation of brain activation signatures. In contrast, as our present results were obtained using speech acts devoid of the indirectness and emotionality confounds, they can unambiguously link speech act type and local cortical activation, that is, fronto–parieto-temporal activation to Requests, and possibly *directive* speech acts in general, as compared with the left parietal activation to Naming, or, possibly *assertive* speech acts more generally (Searle, 1979). The previous studies and the current one agree in that directives activate the fronto-parietal cortex more strongly than assertives. Looking at the previous results discussed above in the context of the present report, the following could be suggested in an attempt at their integration: (i) ToM activation is a general feature appearing across speech act types (cf. right TPJ activation to both Requests and Naming in the present study), but may be amplified for indirect speech acts. (ii) Motor system activation seems more pronounced for directive speech acts than for assertive ones.

### Theory of mind and common ground processing

The general engagement across speech act types of an area belonging to the core ToM network, the right TPJ, seems to be in good agreement with the view that both declarative and Requestive actions represent relevant ways for communicating, and likewise involve understanding of intentions, assumptions and the so-called common ground of communication partners (Tomasello et al., 2007). However, although understanding the intentions and assumptions of the communication partners may be seen as more relevant for Request actions (considering the intention to obtain the Requested object, the assumption that the other person can provide it, and many others) compared with Naming, we did not find any evidence of the difference between speech acts in key regions of the ToM network. Previous results seem to partly contrast with this finding, as studies on communicative pointing have shown that declarative pointing – to inform others about one's own taste – can lead to stronger engagement of ToM and affective-emotion areas than Requestive pointing performed to obtain food (Brunetti et al., 2014, Committeri et al., 2015). However, this virtual discrepancy with the present findings can be explained by task differences. As the ‘declarative’ speech act in this important work by Brunetti and Committeri was made to express a personal taste and preference, it can be seen as closely related to ‘expressive’ speech acts (Searle, 1979) performed to externalise an internal emotional state, i.e., the love for specific food items. The engagement of limbic and ToM circuits in these studies may therefore reflect the emotion-expressive character, rather than the declarative aspect of the ‘expressive–declarative pointing’ actions implemented. In addition, studies that reported ToM activation often involved tasks explicitly requiring subjects to reflect on the communicative intention of utterances or actions (Brass et al., 2007, Spunt and Lieberman, 2013, Van Ackeren et al., 2012). Our observation of speech act independent activity in one part of the ToM network, in the right TPJ (Carter and Huettel, 2013), is consistent with earlier reports that the ToM network is involved in communicative processing of linguistic and nonlinguistic information (Brass et al., 2007, Enrici et al., 2011, Lombardo et al., 2010, Ramsey and Hamilton, 2012, Spunt et al., 2011, Walter et al., 2004). We should, however, mention that the results of our previous MEG study (Egorova et al., 2014), in which single-trial speech acts were presented, suggested preferential engagement of ToM areas in Request understanding, yielding some support for its speech act specificity. In sum, some open questions remain to be addressed in future studies that could investigate the possible specificity of ToM network activations to speech acts, tasks and specific features of experimental paradigms in more detail.

### Referential processing

For both Naming and Requesting of objects, a link must be made between a word and an object. However, as Naming specifically directs the attention of the listener to this referential information, we hypothesized that temporal and left temporo-parietal regions (particularly the angular gyrus) relevant for referential knowledge would be more active during Naming than during Requesting. Our results provide only weak support of this hypothesis, as temporal areas did not show a significant difference between the speech acts, and the AG only revealed a marginally significant effect (Naming > Requesting).

### Hemispheric involvement

**Fig. 3:**
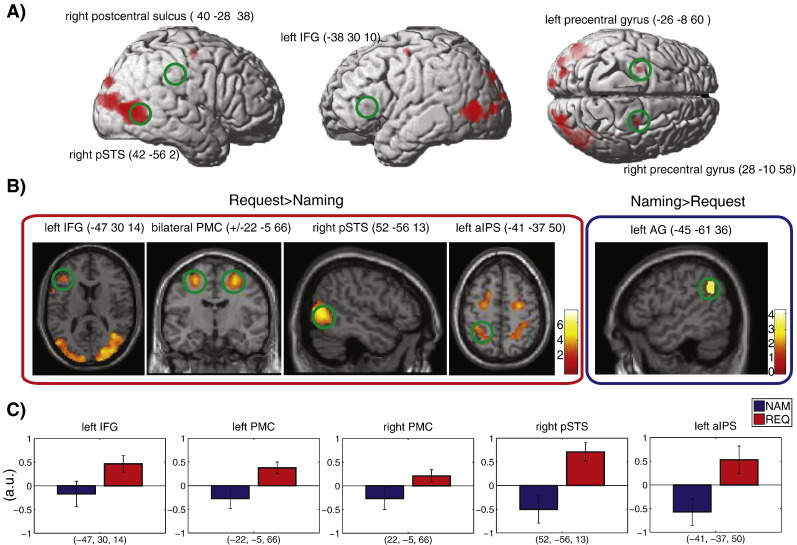
Main results. A. Whole-brain analysis activation for the contrasts “Request > Naming” (shown in red) and “Naming > Request” (in blue), rendered at p < 0.05 FDR-corrected. B. ROI analysis, small volume corrected (SVC) at FWE p < 0.05, rendered at p < 0.01 (unc.), k = 10. C. Signal extraction ROI analysis. pSTS — posterior superior temporal sulcus, IFG — inferior frontal gyrus, aIPS — anterior intraparietal sulcus, vmPFC — ventromedial prefrontal cortex, AG — angular gyrus, PMC — premotor cortex.

Consistent with earlier reports (Weylman et al., 1989, Zaidel et al., 2000), both hemispheres were active in speech act processing, with specific areas in each hemisphere selectively responding to different speech act types. In line with the observation of Soroker et al. (2005), who examined patients with focal cortical lesions and found that lesions in both hemispheres can impair the ability to understand speech acts, we here report that activation in both hemispheres indexes speech act processing. For example, bilateral premotor activations appeared to be stronger for Requests than for Naming. On the other hand, these data do not strongly support the hypothesis put forward in some previous studies (Holtgraves, 2012, McDonald, 2000) that the right hemisphere is more relevant for pragmatic knowledge than the left. Instead, as has previously been suggested for semantic processing (Pulvermüller et al., 2009), pragmatic information about speech acts appears to be carried by neuronal circuits distributed across both cortical hemispheres (Fig. 3A).

## Conclusions

Speech act understanding is reflected in local cortical activity. In participants observing and understanding communicative interactions, in which one actor either Named objects in front of their Partner or Requested objects from that Partner, these different speech acts of Requesting and Naming activated different sets of cortical areas. The main function of Naming is to refer to an object by using a linguistic expression, which requires referential-semantic knowledge linking the two. The left angular gyrus, interfacing between visual and language areas tended to show relatively stronger activity during Naming compared with Requests. In contrast, Request understanding implies forming rich predictions on likely partner actions, which could typically follow this act in social communicative interaction (handing over the object, denying the Request etc.). Consistent with the relevance of predictive action knowledge for this speech act type, Requests activated the inferior frontal, premotor, parietal and temporo-occipital cortices most important for action and action sequence processing and for predicting future action performance and (auditory/visual) perception. The right TPJ, known to be the main site for the processing of theory of mind and common ground knowledge, was found equally active for both speech acts. In sum, we show that the key human capacity of communicative use of language in social interaction context relies on a coordinated effort of a bilaterally distributed network unifying a range of multimodal neurocognitive systems. Whilst only two typical speech act types were examined here, systematic neuro-cognitive investigations of the brain basis of a wide range of communicative actions seems to be an exciting new arena for exploring the unique human capacity of social-communicative interaction.

## Figures and Tables

**Table 1:** Psycholinguistic and semantic stimulus properties. The table shows mean values and standard error of the mean for each psycholinguistic and semantic parameter.

Psycholinguistic and semantic properties of word stimuli	Mean value (SE)
Number of letters	4.2 (0.09)
Word form frequency	25.92 (4.37)
Logarithmic to base 10 of word frequency	1.16 (0.05)
Lemma frequency	58.13 (8.83)
Logarithm to base 10 of lemma frequency	1.51 (0.05)
Orthographic bigram frequency	3611.76 (1983.85)
Orthographic trigram frequency	3604.12 (273.79)
Orthographic neighbourhood size	8.58 (0.67)
Number of meanings	1.31 (0.07)
Word from frequency when used as a noun	25.4 (4.86)
Word from frequency when used as a verb	1.02 (0.4)
Lemma frequency when used as a noun	53.55 (11.7)
Lemma frequency when used as a verb	25.94 (10.38)
Action-relatedness	3.89 (0.12)
Hand-relatedness	3.71 (0.14)
Visual movement-relatedness	4.09 (0.12)
Familiarity	4.95 (0.16)
Imageability	6.45 (0.06)
Concreteness	6.66 (0.05)
Arousal	2.79 (0.11)
Valency	4.33 (0.08)
Potency	3.93 (0.1)

**Table 2:** Whole-brain random effects analysis for the contrast “Words > Faces”. For each region the table shows the label, hemisphere, Brodmann area, MNI coordinates, p-value (uncorrected and FDR corrected), T-value, and Z-score.

Region	Hemisphere	Brodmann area	MNI coordinates			p-Value (unc.)	p-Value (FDR)	T	Z
x	y	z
Superior temporal/Heschl's gyrus	L	48/42	− 48	− 14	− 2	0.000	0.025	5.17	4.43
Superior temporal	R	22	62	− 16	− 2	0.000	0.016	6.28	5.1
Middle temporal	L	21/22	− 64	− 36	8	0.000	0.026	4.95	4.28
Inferior temporal	L	20	− 50	− 32	− 16	0.000	0.039	4.22	3.77
Caudate	L	48	− 22	10	22	0.000	0.026	4.89	4.24
Hippocampus	L	20	− 38	− 22	− 8	0.000	0.045	4.09	3.67
Hippocampus	R	20	42	− 30	− 8	0.000	0.032	4.41	3.9
Angular gyrus	R	39	48	− 60	50	0.000	0.026	4.8	4.18

**Table 3:** Whole-brain random effects analysis for the contrast “Request > Naming”, p < 0.05 FDR-corrected. Regions in bold indicate a priori ROIs. For each region the table shows the label, hemisphere, Brodmann area, MNI coordinates, p-value (uncorrected and FDR corrected), T-value, and Z-score. The reverse contrast “Naming > Request” did not produce any activations that were significant at the FDR-corrected p < 0.05 threshold.

Region	Hemisphere	Brodmann area	MNI coordinates			p-Value uncor	p-Value FDR	T	Z
x	y	z
Middle occipital	L	37	− 42	− 70	0	0.000	0.013	6.33	4.48
**Posterior temporal**	**R**	**37**	**42**	**− 56**	**− 2**	**0.000**	**0.013**	**7.41**	**4.98**
Superior occipital	L	19	− 22	− 88	38	0.000	0.018	4.99	3.86
Superior occipital	L	18	− 20	− 98	22	0.001	0.047	3.91	3.26
Superior occipital	R	18	24	− 90	32	0.000	0.019	4.93	3.83
Superior occipital	L	17	− 12	− 98	18	0.000	0.023	4.76	3.74
**Postcentral**	**R**	**3**	**40**	**− 28**	**38**	**0.001**	**0.047**	**3.92**	**3.26**
**Superior frontal/precentral**	**L**	**6**	**− 26**	**− 8**	**60**	**0.000**	**0.023**	**4.74**	**3.73**
**Superior frontal/precentral**	**R**	**6**	**28**	**− 10**	**58**	**0.000**	**0.025**	**4.65**	**3.68**
**Inferior frontal triangular**	**L**	**46**	**− 38**	**30**	**10**	**0.001**	**0.046**	**3.95**	**3.28**
**Inferior frontal triangular**	**L**	**45**	**− 46**	**28**	**8**	**0.001**	**0.049**	**3.87**	**3.23**

**Table 4:** ROI analysis SVC, corrected at p < 0.05 family-wise error small volume correction (FWE-SVC), 8-mm spheres. For each region the table shows the label, MNI coordinate, p-value_uncor_, p-value_FWE-SVC_, T-value, and Z-score.

ROI label	x	y	z	p uncor	p FWE	T	Z
*Request > Naming*							
Left inferior frontal gyrus triangular, lIFG (AAL)	− 47	30	14	0.001	0.028	3.87	3.23
Left premotor cortex, lPMC (Willems et al., 2010b)	− 22	− 5	66	0.000	0.009	4.56	3.63
Right premotor cortex, rPMC (Willems et al., 2010b)	22	− 5	66	0.001	0.034	3.76	3.16
Right posterior superior temporal sulcus, rpSTS (Van Overwalle and Baetens, 2009)	52	− 56	13	0.000	0.015	4.25	3.46
Left anterior intra-parietal sulcus, laIPS (Van Overwalle and Baetens, 2009)	− 41	− 37	50	0.001	0.039	3.66	3.10
Medial prefrontal cortex, mPFC (Van Overwalle and Baetens, 2009)	1	56	13	–	–	–	–
Left temporo-parietal junction, lTPJ (Van Overwalle and Baetens, 2009)	− 52	− 55	29	–	–	–	–
Right temporo-parietal junction, rTPJ (Van Overwalle and Baetens, 2009)	52	− 55	29	–	–	–	–
Left anterior cingulate, lACC (AAL)	− 5	35	14	–	–	–	–
Right anterior cingulate, rACC (AAL)	7	37	16	–	–	–	–

*Naming > Request*							
Left angular gyrus, lAG (AAL)	− 45	− 61	36	0.002	0.071	3.27	2.84

## Footnotes

1 In this study we concentrated on the analysis of identical single word utterances representing the speech acts of Naming and Requesting, which were introduced by the Context sentences. Since each speech act, as represented by the action sequence tree, can be followed by certain other types of speech acts, it is possible that the upcoming speech act type could be predicted already at the stage of the Context sentence itself. For example, the speech act of Inquiry (“What are these called?”) can be typically followed by Naming (“Tea”), Clarification (“What did you say?”), and Rejection (“I don't know”), whilst the speech act of Offer (“What can I get you?”) could be preferentially followed by Requesting (“Tea”), Clarification (“What do you have?”), and Rejection (“Nothing”), respectively. In order to prevent predictability of upcoming Naming or Requesting actions, we included these alternative speech acts in our study, as well as trials with still faces replacing the word videos (see Fig. 1). Our study did not have sufficient power to examine the contrast between Context sentence types; it is possible that some precursors of speech act differences could be detected before the onset of the critical words.
